# Rapid Retrieval of Lung Nodule CT Images Based on Hashing and Pruning Methods

**DOI:** 10.1155/2016/3162649

**Published:** 2016-11-22

**Authors:** Ling Pan, Yan Qiang, Jie Yuan, Lidong Wu

**Affiliations:** ^1^College of Computer Science and Technology, Taiyuan University of Technology, Taiyuan 030024, China; ^2^Shanxi Provincial People's Hospital, Taiyuan 030012, China; ^3^University of Texas at Tyler, 3900 University Blvd., Tyler, TX 75799, USA

## Abstract

The similarity-based retrieval of lung nodule computed tomography (CT) images is an important task in the computer-aided diagnosis of lung lesions. It can provide similar clinical cases for physicians and help them make reliable clinical diagnostic decisions. However, when handling large-scale lung images with a general-purpose computer, traditional image retrieval methods may not be efficient. In this paper, a new retrieval framework based on a hashing method for lung nodule CT images is proposed. This method can translate high-dimensional image features into a compact hash code, so the retrieval time and required memory space can be reduced greatly. Moreover, a pruning algorithm is presented to further improve the retrieval speed, and a pruning-based decision rule is presented to improve the retrieval precision. Finally, the proposed retrieval method is validated on 2,450 lung nodule CT images selected from the public Lung Image Database Consortium (LIDC) database. The experimental results show that the proposed pruning algorithm effectively reduces the retrieval time of lung nodule CT images and improves the retrieval precision. In addition, the retrieval framework is evaluated by differentiating benign and malignant nodules, and the classification accuracy can reach 86.62%, outperforming other commonly used classification methods.

## 1. Introduction

The early diagnosis and treatment of lung cancer patients can help improve their survival rate [1]. However, with the development and improvement of various medical image scanning technologies, especially computed tomography (CT), the number of medical images is growing explosively every year. Hence, in the early medical screening process, reviewing lung lesions is an extremely labor-intensive job for radiologists. In addition, when reviewing and analyzing lesions, radiologists mainly rely on their diagnostic experience, and the diagnosis tends to be highly subjective. Moreover, clinical statistical studies show that the same radiologist, at different times, under different states of physical fatigue, may come up with a different diagnosis for the same CT image. Therefore, it is necessary to retrieve similar lung nodule CT images to improve diagnostic efficiency. By obtaining similar images from a CT image repository of pulmonary nodules, the anamnesis and successful treatments of these images can be viewed as clinical references for the case under consideration, which can lessen the reliance on a physician's clinical diagnostic experience to a certain degree.

Given the explosive growth of the number of current lung images and advantage of medical image retrieval for physicians' diagnosis of lung lesions, in this paper, a novel retrieval framework based on a hashing and pruning algorithm for lung nodule CT images is proposed. When retrieving similar lung nodule CT images, it not only can reduce the memory space required but also further shorten the retrieval time and improve precision with a pruning-based similarity measure method.

The remainder of this paper is organized as follows.  Section 2 introduces previous work related to lung nodule image retrieval and current popular retrieval methods.  Section 3 describes the proposed retrieval framework in detail. Experimental results are presented with some discussion in Section 4.  Section 5 concludes the paper and discusses future work.

## 2. Related Work

Recent years have witnessed the growing popularity of medical image retrieval, and there are many significant results in the field of lung imaging. Oliveira and Ferreira [2] proposed a bag-of-tasks method combining texture features and registration techniques to retrieve lung cancer images. Ng et al. [3] presented an improved hierarchical spatial descriptor and binary descriptor to retrieve similar lung nodule CT images from the perspective of spatial similarity. Aggarwal et al. [4] studied the detection and classification of lung nodules with content-based medical image retrieval. However, given the large number of lung CT images generated every year, effective medical image retrieval is still a difficult challenge.

At present, hashing-based methods for image retrieval can solve the storage and efficiency problems that traditional image retrieval methods may encounter [5]. Further, these hashing methods can transform high-dimensional image data into a low-dimensional binary space by utilizing the constructed hash functions [6]. It is precisely because of these advantages that many scholars focus on researching hashing-based image retrieval technology. Locality-sensitive hashing [7], a pioneering work, can generate compact binary codes with a random threshold. Further, in many hashing methods, such as those in [8–10], principal component analysis (PCA) is a common method for preprocessing image data. The simplest of these approaches is PCAH: after using PCA to reduce the dimensionality of the image data, “0” is viewed as the boundary, and both sides, respectively, correspond to “0” and “1.” Moreover, according to whether label information is used to construct hash functions, hashing methods can be categorized as unsupervised hashing [8, 9, 11], semisupervised hashing [10], or supervised hashing [12, 13]. Additionally, the core of these hashing methods is the minimization of the error when translating the image data into binary space.

Although many hashing and improved hashing methods have been presented, only a few researchers have applied them to medical image retrieval. Liu et al. [14] presented an image retrieval framework for digital mammograms with anchor graph hashing and improved its search accuracy by fusing different features. Jiang et al. [15] used a joint kernel-based supervised hashing algorithm with a small amount of supervised information to compress breast histopathological images into 10-bit hash codes and identified actionable and benign tumors based on the retrieval results. Zhang et al. [16] built a histopathological image retrieval framework using a supervised hashing with kernel (KSH) method and validated the retrieval performance on breast microscopic tissues images.

In our proposed retrieval method for lung nodule CT images, partitioning the dataset with a clustering algorithm is the foundation of the pruning algorithm. The KSH method is then used to translate the images in each cluster into short hash codes and form the hash code database. During retrieval, a pruning algorithm is employed to shorten the retrieval area and further improve the retrieval speed and precision. We use other state-of-the-art hashing methods to validate the proposed pruning algorithm and compare it with other commonly used classification methods to demonstrate the performance of our retrieval framework.

## 3. Description of the Retrieval Framework and Pruning Algorithm

**Figure 1:**
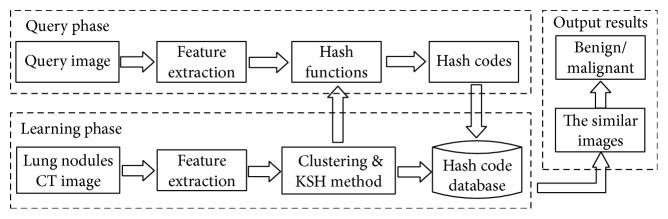
Retrieval framework for lung nodule CT images with hashing and pruning algorithms.

The retrieval framework for lung nodule CT images proposed in this paper consists of two main parts, the learning phase and query phase, as shown in Figure 1. The aim of the learning phase is to build a hash code database. First, we use the extracted visual and medical features to represent each lung nodule CT image. Binary codes are then obtained and stored in a hash code database using the KSH method. In the query phase, given a query lung nodule CT image, we first extract the same features that were extracted in the learning phase and translate them into binary code with the constructed hash functions. Next, similar images are retrieved from the hash code database while using the pruning algorithm. The retrieval results can be used to recognize benign or malignant nodule.

### 3.1. Lung Nodule Feature Extraction

Feature extraction plays an important role in image retrieval and can transform high-dimensional nodule images into a lower-dimensional space while retaining the essential content of the image. Good features can help physicians to distinguish lung nodules efficiently [17–19]. To facilitate analysis and research on lung lesions, we extract lung nodule features based on grayscale, morphology, and texture.

Grayscale features are the most basic characteristics of an image of lung nodules, and the grayscale difference can highlight the corresponding organization and structure. The proposed method extracts three gray level characteristics, grayscale mean, variance, and entropy, where grayscale entropy reflects the grayscale information contained in the nodule image, and is defined as

(1) $$\begin{array}{c} H=-\overset{k-1}{\underset{i=0}{\sum }}H\left(\;i\;\right)\log H\left(\;i\;\right), \end{array}$$

where *H*(*i*) represents the probability density of a different greyscale value and *k* is the number of available gray level values.

Morphological features are the most intuitive visual features and are helpful for identifying tumors. We describe the morphological features of lung nodule mainly using invariant moments, medical signs, and geometric features. We employ the seven invariant moments proposed by Hu to describe the shape characteristics of pulmonary nodules. The calcification area, calcification degree, cavitary area, and cavitary ratio are calculated and represent the medical sign information of the lung nodules. The geometric features consist of lung nodule perimeter, area, maximum diameter, rectangle, and roundness, where roundness describes the degree of deviation of the nodule region from a circular shape, defined as

(2) $$\begin{array}{c} F=\frac{4\pi A}{L^{2}}, \end{array}$$

where *A* is the area of the nodule region and *L* is the perimeter.

Texture features can provide important information on the health of the lung. For example, the structure of diseased tissue is more chaotic and rough than healthy tissue [18]. Here, the gray level co-occurrence matrix is used to extract texture features. This is the most widely used texture analysis method in medical imaging. The computed features include 14 characteristic values, such as contrast, angular second moment, entropy, and inverse difference moment, which are defined as

(3) CON=∑n=0k−1n2∑i−j=nGi,j,ASM=∑i=0k−1 ∑j=0k−1Gi,j2,ENT=−∑i=0k−1 ∑j=0k−1Gi,jlog⁡Gi,j,IDM=−∑i=0k−1 ∑j=0k−1Gi,j1+i−j2.

We also calculate the mean and variance of these 14 feature values.

A detailed description of the extracted multiple features is given in Table 1. By extracting the lung nodule features from grayscale, morphology, and texture, we utilized a 104-dimensional feature vector to uniquely represent each lung nodule CT image, constructed as follows:

(4) x=f1,f2,f3︸gray,f4,…,f10,f11,…,f15,f16,…,f20︸morphology,f21,f22,…,f104︸texture.

### 3.2. Building the Hash Code Database

**Figure 2:**
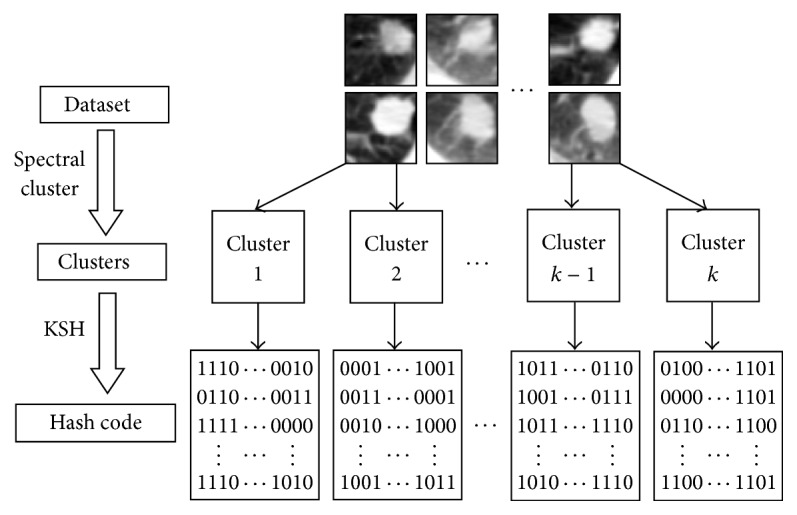
Hash code database construction.

In order to achieve the rapid retrieval of lung nodule CT images using the proposed pruning algorithm, it is necessary to partition the dataset before constructing the hash code database using the hashing method. As shown in Figure 2, the construction of a hash code database includes two parts: clustering and hashing. In the first part, a spectral clustering algorithm is used to partition our training dataset into several clusters so that the distribution of lung nodule CT images in each cluster is near uniform. In addition, when retrieving a query image, the retrieval scope can be narrowed according to the distance between the query image and cluster centers. In the second part, the KSH method is employed to construct hash functions for the whole training dataset and obtain the hash code database. Furthermore, the uniform distribution of lung nodule CT images in clusters could reduce the instability of retrieval performance caused by the uneven distribution of images.

#### 3.2.1. Partitioning the Lung Nodule Dataset

Spectral clustering is a clustering algorithm based on spectral graph theory that can identify a sample space with arbitrary shapes and converge to the global optimal solution. Furthermore, the obtained clustering results outperform traditional clustering approaches, such as *k*-means or single linkage clustering [20, 21].

In this paper, given a training dataset of lung nodule CT image *χ* = {*x*_1_,…, *x*_*n*_} ∈ *R* ^*d*^, where *n* is the number of images and *d* is the dimension of extracted features, a graph *G* is first built to represent these data. The vertices *V* in the graph represent lung nodule CT images, and the weights of edges *E* represent the similarity of any two lung nodule CT images. An undirected weighted graph *G* = (*V*, *E*) based on the similarity of the images then can be obtained. Thus, the clustering problem is converted into a graph partitioning problem on *G*.

The main step of spectral clustering is to construct graph partitions based on graph Laplacian matrix *L*. Here, we use the normalized Laplacian matrix, which is defined as

(5) $$\begin{array}{c} L=D^{-1/2}\left(\;D-W\;\right)D^{-1/2}, \end{array}$$

where *W* is a similarity matrix, defined as

(6) $$\begin{array}{c} W_{ij}=\exp^{-{\left\|x_{i}-x_{j}\right\|}^{2}/2\delta^{2}}\;\;, \end{array}$$

where *δ* is a parameter and *D* is a diagonal matrix obtained from *W*, defined as

(7) $$\begin{array}{c} D_{ij}=\left\{\begin{array}{cc} \overset{n}{\underset{j=1}{\sum }}W_{ij}, & i=j\\ 0, & i\neq j. \end{array}\right. \end{array}$$

The classical *k*-means method is then utilized to cluster the eigenvectors of Laplacian matrix *L*. Using the above steps, we can acquire several clusters in which the lung nodule CT images are similar to each other. This is the foundation for achieving a pruning algorithm and is helpful for improving the retrieval speed as well as precision.

#### 3.2.2. Construction of Hash Functions

One of the factors affecting the performance of a hashing method is the ability to preserve the similarity of any two images in the original feature space. Hence, the key to a hashing method is to construct appropriate hash functions and maintain the similarity within the hash code. KSH is a supervised hashing method that uses a limited amount of supervised information for learning hash functions, and the retrieval results are better than other unsupervised hashing methods as well as some supervised hashing methods.

Given all the lung nodule CT images in training dataset *χ*, we need to construct a group of hash functions *H* = {*h*_1_(*x*),…, *h*_*r*_(*x*)}, each of which will generate a single hash bit. In addition, if the length of the hash code is *r*, then *r* hash functions will be constructed. A hash function is defined as

(8) $$\begin{array}{c} h\left(\;x\;\right)=sgn\left(\;f\left(\;x\;\right)\;\right)=sgn\left(\;\overset{m}{\underset{j=1}{\sum }}\kappa \left(\;x_{\left(j\right)},x\;\right)a_{j}-b\;\right), \end{array}$$

where *κ*(*x*, *y*) = exp⁡(−‖*x* − *y*‖/2*σ* ^2^)is a Gaussian kernel function for solving the problem of the linear inseparability of lung nodule images, {*x*_(1)_,…, *x*_(*m*)_} are samples randomly selected from the training dataset *χ* to support kernel computation, {*a*_1_,…, *a*_*m*_} are a group of coefficients, sgn(*x*) is a sign function outputting 1 for positive input and −1 for negative input, and *b* ∈ *R* is a bias defined as

(9) $$\begin{array}{c} b=\frac{1}{n}\overset{n}{\underset{i=1}{\sum }}\;\overset{m}{\underset{j=1}{\sum }}\kappa \left(\;x_{\left(j\right)},x_{i}\;\right)a_{j}. \end{array}$$

**Figure 3:**
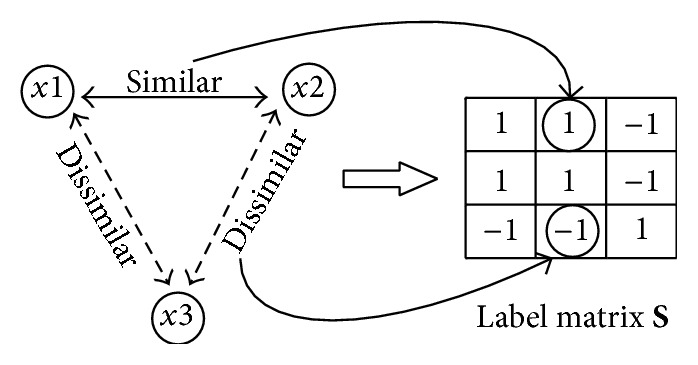
Construction of a label matrix.

As the differences in lung nodule images are not apparent, as they are in natural images, the coefficient vector **a** = [*a*_1_,…,*a*_*m*_]^*T*^ is vital for generating distinguishable hash functions. Here, supervised information, that is, a label matrix, is utilized to solve this problem. During the image preprocessing, we mark each lung nodule CT image with a label 1 or 0 based on whether it shows benign or malignant lesions. The *l*  (*m* < *l* ≤ *n*) images from the training dataset are then randomly selected to construct a label matrix **S** ∈ *R* ^*l*×*l*^. The construction process is shown in Figure 3.

When retrieving a query image, the Hamming distance is a commonly used method to measure the similarity between the query image and the database images. However, it is difficult to directly compute this distance because of its complex formula. The research in [13] explains the corresponding relation between the Hamming distance and code inner product. Hence, the objective function can be defined using a code matrix formed by the selected *l* samples and label matrix to solve **A** = [**a**_1_,…, **a**_*r*_] for hash functions *H* as follows:

(10) $$\begin{array}{c} \underset{A}{\min}\;Q\left(\;A\;\right)={\left\|\;\sum_{k=1}^{r}sgn\left(\;{\overset{-}{K}}_{l}a_{k}\;\right){\left(\;sgn\left(\;{\overset{-}{K}}_{l}a_{k}\;\right)\;\right)}^{T}-rS\;\right\|}_{F}^{2}, \end{array}$$

where ‖·‖_*F*_ is the Frobenius norm and ${\overset{-}{K}}_{l}$ represents the kernel computation for the *l* images involved in label matrix **S** and is expressed as
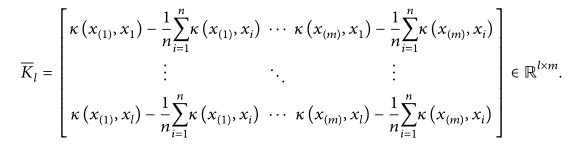
(11)


Thus, by minimizing the error between the code matrix and label matrix, the hash functions with supervised information can be acquired and used to encode each lung nodule CT image.

### 3.3. Retrieval Process for Lung Nodule CT Images

**Figure 4:**
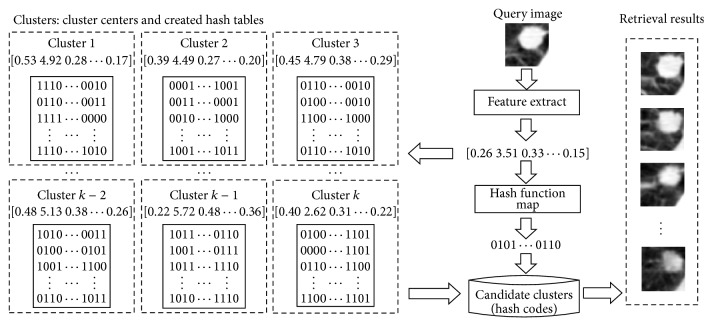
Procedure of lung nodule CT image retrieval with pruning.

The aim of the proposed method is to achieve rapid retrieval for lung nodule CT images with higher precision. Hence, a pruning algorithm is presented. The retrieval procedure with pruning algorithm is illustrated in Figure 4.

Given a query image, the retrieval process includes the following three steps: (1) determining candidate clusters, that is, selecting some clusters as the candidate clusters to which the query image may belong, (2) encoding the query image, that is, compressing the extracted relevant features into binary codes with the constructed hash functions, and (3) calculating similarity, that is, computing and sorting the code inner products between the query image and images in the candidate clusters and returning the similar images according to the similarity.

However, when sorting the code inner products, if the length of hash code is *r*, the value range of code inner product is [−*r*, *r*], and it is impossible to directly sort the images that have the same code inner products. In order to solve this problem, we designed a decision rule: compare the distances between the query image and the clusters that these similar images belong to and return the image with a smaller distance. The whole pruning process is shown in Algorithm 1.

**Algorithm 1 (pruning algorithm for image retrieval).**

 

*Input.* Query lung nodule image *q*, cluster centers {*μ*_1_,…, *μ*_*k*_}, number of candidate clusters *m*  (0 < *m* < *k*), hash functions *H* = {*h*_1_(*x*),…, *h*_*r*_(*x*)}, hash codes {code_*μ*_1__,…, code_*μ*__*k*_} in clusters, and number of returned similar images *p*.

*Output.* Similar images {*x*_1_,…, *x*_*p*_}.

**Step 1 .**

Calculate the distance between *q* and each cluster center using

(12) $$\begin{array}{c} d={\left\|\;q-\mu_{i}\;\right\|}^{2},\;\left(i=1,\ldots ,k\right). \end{array}$$

**Step 2 .**

Rank and select the top *m* clusters as the candidate clusters using

(13) $$\begin{array}{c} cluster_{1},\ldots ,cluster_{m}⟵sort\left(\;d_{i},\text{“abscend"}\;\right). \end{array}$$

**Step 3 .**

Compress the query image into code with *H*.

**Step 4 .**

Calculate the code inner products between *q* and the images in the candidate clusters as follows:

(14) $$\begin{array}{c} sim=code\circ code_{\mu_{i}},\;\left(i=1,\ldots ,m\right). \end{array}$$

**Step 5 .**

Rank the code inner products sim as follows:

(15) $$\begin{array}{c} x_{1},\ldots ,x_{p}⟵sort\left(\;sim,\text{“descend"}\;\right). \end{array}$$

**Step 6 .**

If equal (sim_*i*_, sim_*j*_), then compare the corresponding distances *d*_*i*_ and *d*_*j*_ in Step 1.

**Step 7 .**

If *d*_*i*_ > *d*_*j*_, then return image *x*_*j*_ first.

**Step 8 .**

Repeat Steps 6-7 until *p* similar images are returned in order.

## 4. Experimental Results and Discussion

In this section, we first introduce the dataset used in the evaluation and the extracted multiple features. Next, we discuss the parameter settings in our proposed retrieval method. We then validate our pruning algorithm on different hashing methods. Finally, we evaluate the performance of our retrieval framework by comparing it with other commonly used classification methods. All our experiments were implemented in MATLAB R2014b on a workstation with Intel Core i7-4770 CPU 3.40 GHz and 8 GB of RAM.

### 4.1. Image Dataset

The image data used in our experiments are from the LIDC dataset [22]. The LIDC dataset contains 1,018 cases, each of which includes a set of chest CT images and an associated XML file that records some relevant information about the lung nodules (such as whether they are benign or malignant). There are a total of 7,371 nodules labeled at least by one radiologist and 2,669 of these nodules are marked “nodule ≥ 3 mm.” Here, in order to ensure that the training dataset does not influence the testing dataset (i.e., that no images belonging to the same case appear in both the training dataset and the testing dataset), the dataset in this research is constructed from 600 cases. Further, we randomly selected 70% of them as training data and the remaining 30% as testing data. Here, slices with unclear nodules in each case were discarded, resulting in a total of 2,450 slices in our dataset. The detailed contents of this dataset are listed in Table 2.

**Figure 5:**
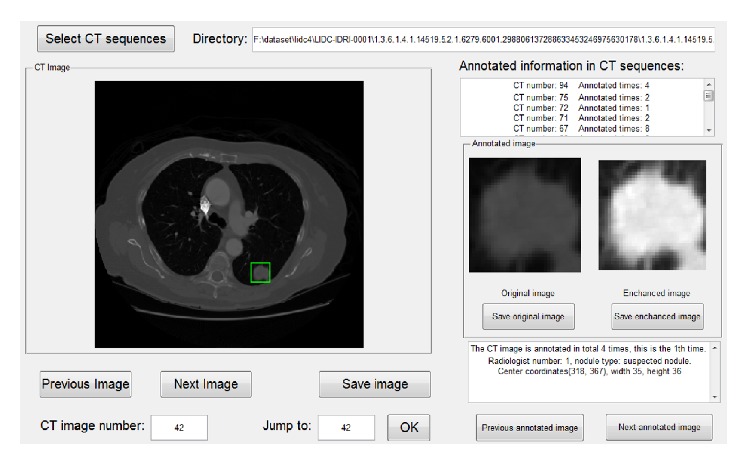
Visual interface for obtaining the relevant information from the lung nodule image.

This study is aimed at lung nodules, so the first step in our experiment is to obtain the region of interest. As the XML files in the LIDC record information about the lung nodules, our team designed a visual interface and parsed these XML files to obtain the relevant information, as shown in Figure 5. The rectangular regions containing lung nodules were extracted and viewed as regions of interest. Thus, the lung image database was constructed based on these regions.

In order to facilitate the research and analysis of lung lesions, we extracted the multiple features of lung nodules and stored them into our database in advance.  Table 3 describes some feature values extracted from the lung nodule images.

### 4.2. Parameter Settings

There are three main parameters affecting the performance of the proposed retrieval framework. They are the length of the compact hash code* bit*, number of clusters *k*, and number of candidate clusters used for retrieval *m*.

The effect of a hashing method is to transform the high-dimensional image features of lung nodules into a low-dimensional hash code to represent each image. Additionally, based on the experience of a large number of studies, the length of hash codes in this paper is set to between 8 and 64 bits. Here, in order to determine the influence of the hash code length on the retrieval results, we first did our experiment using the KSH method only (without using the pruning algorithm). The retrieval precision for different hash code lengths is reported in Table 4, and the best retrieval results are obtained when* bit* = 48. Retrieval precision is one of the criteria used to evaluate the performance of a retrieval method and defined as

(16) $$\begin{array}{c} precision=\frac{relevant\_results}{results}, \end{array}$$

where results indicate the number of returned images and relevant_results denote the number of correct results in the returned images as judged by the label information.

Next, setting* bit* to 48, we discuss how to set parameters *k* and *m* appropriately to achieve a retrieval precision of 85.42% within the shortest retrieval time. Retrieval time refers to the period of time beginning with the encoding of the test images and ending when the similar lung nodule CT images have been obtained.

**Figure 6:**
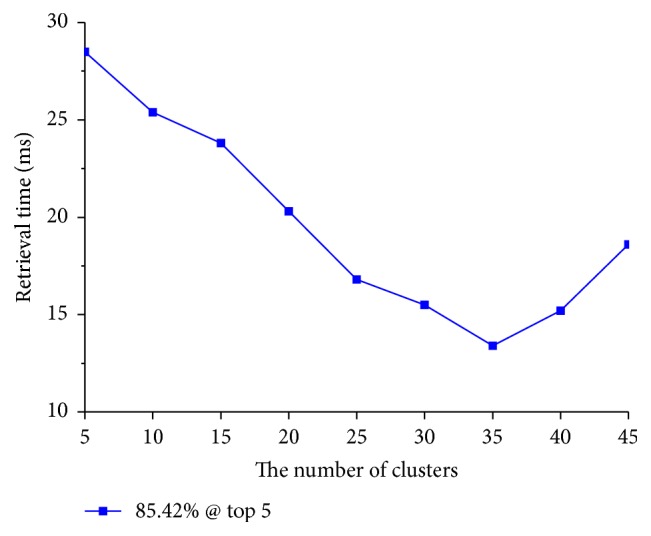
Retrieval time with respect to number of clusters for* bit* = 48.

In order to reach the same retrieval precision without using a decision rule, the values of parameters *m* and *k* were acquired through experiment and are shown in Table 5.  Figure 6 demonstrates how the retrieval time changes according to the number of clusters when the length of hash code is 48 bits. We can see that, as the number of clusters increases, the retrieval time decreases when *k* < 35, but when *k* > 35, the retrieval time increases. Considering Table 5 and Figure 6, the reason for this situation is that as the number of clusters increases, the reduced number of images in the candidate clusters is greater than the increased number of images in the newly produced candidate clusters. That is, the total number of images in the candidate clusters needed to reach the same retrieval precision is less than before. However, when *k* > 35, the situation is reversed. This may be similar to the phenomenon of overfitting in statistical learning. When the number of clusters increases, the retrieval result may be worse. Moreover, as the number of clusters increases, the time required to calculate the distance between the query image and each cluster center cannot be ignored. Hence, in order to obtain a better retrieval result, we set the length of the hash code to 48 bits and the number of clusters to 35. Additionally, as shown in Table 5, the number of candidate clusters is set to eight.

**Figure 7:**
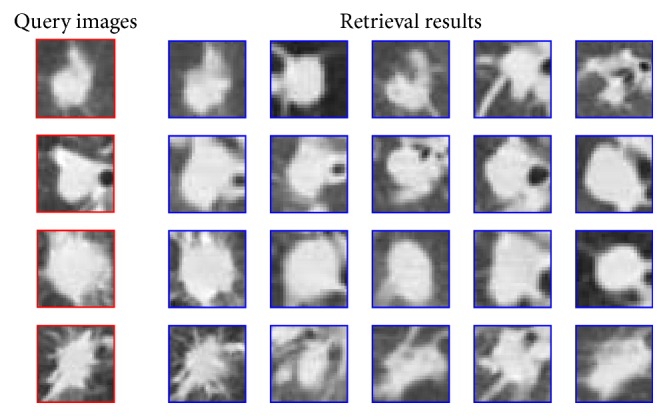
Retrieval results for lung nodule CT images using the proposed method.

The retrieval results of lung nodule CT images based on the above parameter settings are shown in Figure 7, where the first two query images are malignant tumors, and the last two are benign tumors.

### 4.3. Performance Comparison of the Pruning Algorithm

**Figure 8:**
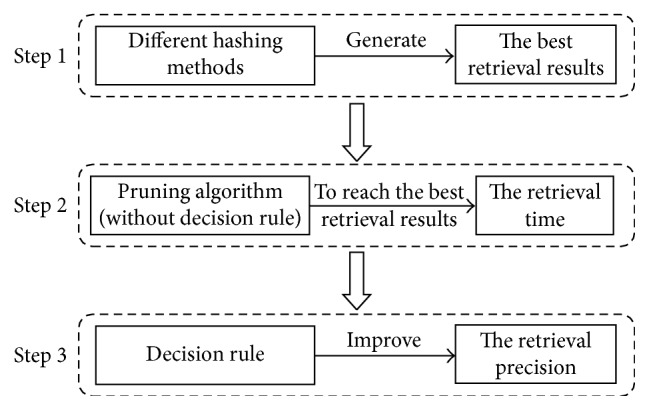
Experiment flow for evaluating the performance of the pruning algorithm.

In these experiments, we applied the proposed pruning algorithm to different hash methods such as kernelized locality-sensitive hashing (KLSH) [11], spectral hashing (SH) [8], binary reconstructive embedding (BRE) [12], PCAH, and iterative quantization (ITQ) [9]. We compared the retrieval time and precision to evaluate the performance of the pruning algorithm. The experiment flow is shown in Figure 8.

**Figure 9:**
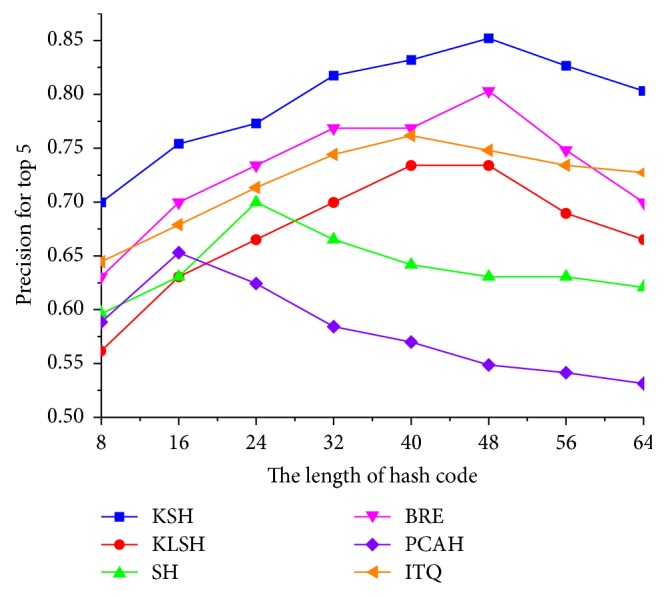
Retrieval precision of different hashing methods for different hash code lengths (without using the pruning algorithm).

First, the different hashing methods were utilized to retrieve the similar lung nodule CT images without using the pruning algorithm.  Figure 9 shows the retrieval precision of these hashing methods, in which the retrieval result of the KSH method outperforms the other hashing methods.

**Figure 10:**
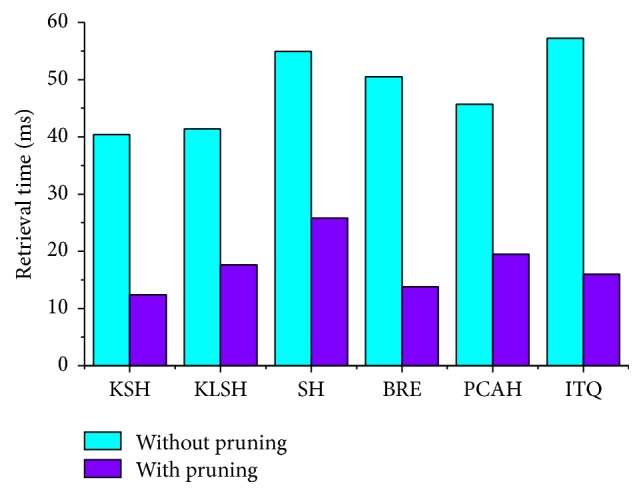
Comparison of different hashing methods with and without the pruning algorithm in terms of retrieval time.

We then applied the proposed pruning algorithm (without using the decision rule) to these hashing methods and validated its performance when the dataset is partitioned into 35 clusters. The parameter settings for the hash code length and number of candidate clusters are shown in Table 6, which differ depending on the highest precision that these hashing methods can reach.  Figure 10 shows the retrieval time for all query images for the different hashing methods. We can see that the retrieval speed of these hashing methods using the pruning algorithm is about 2–4 times faster than when these methods do not use the pruning algorithm. Hence, the proposed pruning algorithm clearly reduces the retrieval time of lung nodule CT images.

**Figure 11:**
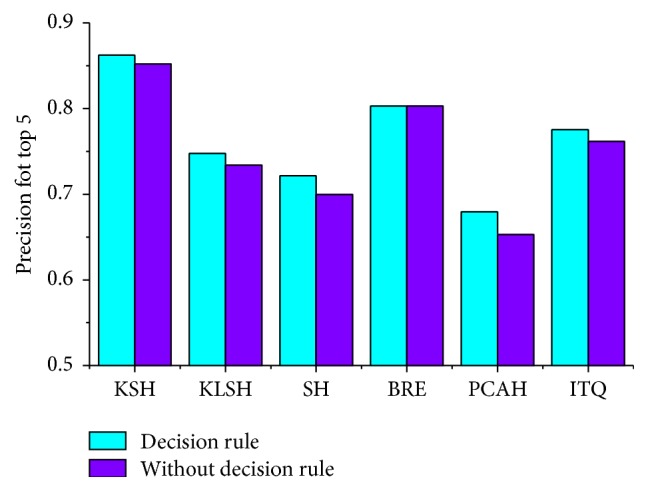
Influence of decision rule on retrieval precision.

Moreover, the decision rule designed in the pruning algorithm is also helpful for improving the retrieval precision to some extent. By comparing the distance between the query image and the clusters, the most similar lung image can be returned first.  Figure 11 shows the influence of the decision rule on the highest retrieval precision of different hashing methods.

### 4.4. Evaluation of the Retrieval Framework

**Figure 12:**
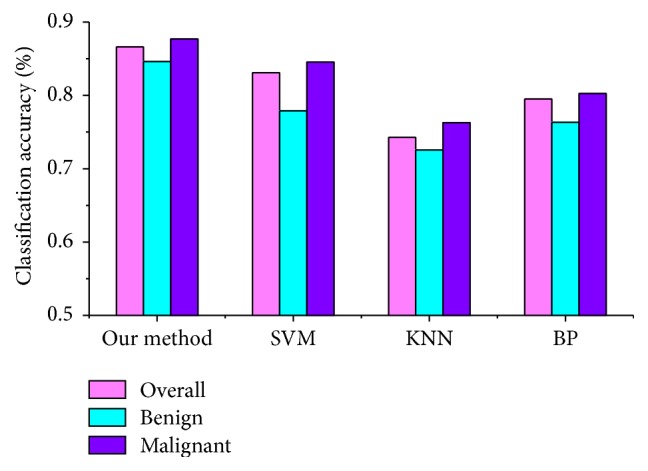
Comparison of the accuracy of different classification methods.

Figure 12 compares different classification methods, the support vector machine (SVM), back propagation (BP), and *K* nearest neighbors (KNN), with our hashing-based method, using the classification accuracy of benign and malignant lesions. In this evaluation, we compared them with respect to overall (all nodules in the test dataset), benign nodule, and malignant nodule accuracy in the test dataset. We judged whether the query lesion was benign or malignant using the returned similar lung nodule CT images. The judging method is similar to the idea of the KNN algorithm, that is, if the number of benign lung nodules is greater than the number of malignant lung nodules in the returned similar images, the query image is diagnosed as a benign tumor. Otherwise, the query tumor is diagnosed as malignant.

The KNN algorithm is always used as a baseline classical classification method in machine learning. Here, we employ the Euclidean distance to obtain similar samples and set *K* to 5. However, the calculation is not efficient enough for the high-dimensional image features. Our hashing method leverages the compact hash code and code inner products to measure the similarity, which is helpful for improving its efficiency. The BP neural network is one of the most widely used neural network models. The learning rate is set to 0.01, and the number of iterations is 500 in our experiments. A SVM is a supervised learning model that uses supervised information to bridge the semantic gap. Hence, the classification results of SVM with a radial basis function are better than KNN and the BP method.

Furthermore, we can see that our method significantly outperforms the other three classification methods. The overall classification accuracy can reach 86.62%, with accuracies of 84.61% for the benign lesions and 87.67% for the malignant lesions. This improvement illustrates that hash functions with supervised information actually preserve the similarity of the images in the original feature space and validate its retrieval performance.

## 5. Conclusion

In this paper, in order to improve the retrieval efficiency of lung nodule CT images, we presented a retrieval framework based on the KSH method and a pruning algorithm. Specifically, a clustering method is first used to partition the dataset into several clusters. The KSH method is then utilized to compress the high-dimensional feature vectors into compact hash codes. Finally, a pruning algorithm is employed to narrow the retrieval range and further shorten the retrieval time while improving the retrieval precision. Here, the hash functions are used to map a 104-dimensional image feature of lung nodules into a 48-bit binary code, which, to some extent, reduces the memory space. Low memory cost, fast query speed, and a higher precision demonstrate the suitability of the proposed retrieval framework for lung nodule image retrieval. However, in this paper, we only handle benign and malignant lung nodules, which is a relatively easy task. In future work, the method should be further refined to retrieve lung images at the level of medical signs (such as calcification, lobulation, and speculation) with a higher retrieval precision, helping physicians make reliable diagnostic decisions for clinical cases.

## Figures and Tables

**Table 1:** Description of lung nodule feature extraction.

Feature	Descriptors	Representation
Gray	3 gray features (gray mean, variance, and entropy)	*f*_1_, *f*_2_, *f*_3_
Morphology	7 invariant moments features (Hu moment invariants)	*f*_4_,…, *f*_10_, *f*_11_,…, *f*_15_, *f*_16_,…, *f*_19_, *f*_20_
	5 geometrical features (perimeter, area, roundness, rectangularity, and maximum diameter)	
	4 medical signs (calcification area and degree, cavitary area, and degree) and Fourier descriptor	
Texture	14 texture features from GLCM (contrast, angular second moment, entropy, inverse difference moment, etc.) with 4 different angles as well as the mean, variance of them	*f*_21_, *f*_22_,…, *f*_104_

**Table 2:** Distribution of cases and slices over the lung tumor types involved in the dataset.

Lung tumor type	Cases	Slices
Benign	250	1054
Malignant	350	1396

**Table 3:** Quantitative results of some features of lung nodule images.

Image	Gray		Morphology							Texture (0°)		
			7 invariant moments		Medical signs		Geometrical features					
	Mean	Entropy	*M*_1_	*M*_2_	Calcification degree	Cavitary ratio	Area	Roundness	Rectangle	ASM	CON	IDM
*I*_1_	0.5327	4.9199	0.2789	0.0025	0.1720	0	343	0.3961	0.5489	0.0521	1.3518	0.7151
*I*_2_	0.5403	4.8428	0.2731	0.0016	0.1954	0.2483	302	0.3503	0.5243	0.0451	1.9339	0.6360
*I*_3_	0.4496	4.7236	0.3173	0.0032	0.2268	0	529	0.6274	0.3701	0.0854	2.1015	0.6853
*I*_4_	0.3933	4.4967	0.3757	0.0037	0	0.1985	297	0.5632	0.4127	0.1415	1.5383	0.7105
*I*_5_	0.5293	5.1302	0.2707	0.0017	0.2571	0.1684	412	0.5279	0.2953	0.0267	1.7759	0.6369

**Table 4:** Retrieval precision for the top 5 similar lung images for different hash code lengths.

Bits	8	16	24	32	40	48	56	64
Pre. for top 5	0.6997	0.7541	0.7731	0.8176	0.8351	0.8542	0.8207	0.8031

**Table 5:** Settings for parameters *k* and *m* to reach the retrieval precision at 48 bits.

Clusters *k*	5	10	15	20	25	30	35	40	45
Candidate clusters *m*	2	4	5	6	6	7	8	12	16

**Table 6:** Parameter settings of the different hash methods for the pruning algorithm evaluation.

Parameters	Different hash methods					
	KSH	KLSH	SH	BRE	PCAH	ITQ
*Bit*	48	40	24	48	16	40
*m*	8	9	11	7	9	8
